# Free Fetal Haemoglobin in Severe Early‐Onset Fetal Growth Restriction: A Prospective Multi‐Centre Study

**DOI:** 10.1111/1471-0528.18104

**Published:** 2025-02-19

**Authors:** Adam Brook, Georgia Baynes, Jonathan Scargill, Angelos Evangelinos, Charlotte Brennan‐Richardson, Freya Dow, Yuval Ginsberg, Tal Weissbach, Jana Brodszki, Eva Hansson, Anke Diemert, Kurt Hecher, Katarzyna Maksym, Neil Marlow, Rebecca N. Spencer, Anna L. David, Stefan R. Hansson, Paul Brownbill

**Affiliations:** ^1^ Maternal and Fetal Health Research Centre University of Manchester Manchester UK; ^2^ Manchester Academic Health Sciences Centre Manchester UK; ^3^ Northern Care Alliance NHS Group Royal Oldham Hospital Manchester UK; ^4^ Department of Obstetrics and Gynecology Rambam Medical Centre Haifa Israel; ^5^ Department of Obstetrics and Gynecology Sheba Medical Center Tel Aviv Israel; ^6^ Department of Obstetrics and Gynecology, Institute of Clinical Sciences Lund Lund University and Skåne University Hospital Malmö Sweden; ^7^ Department of Obstetrics and Fetal Medicine University Medical Center Hamburg‐Eppendorf Hamburg Germany; ^8^ Elizabeth Garrett Anderson Institute for Women's Health, 2nd Floor Medical School Building University College London London UK; ^9^ LIGHT Laboratories, Leeds Institute of Cardiovascular and Metabolic Medicine University of Leeds Leeds UK; ^10^ National Institute for Health and Care Research University College London Hospitals Biomedical Research Centre London UK

**Keywords:** alpha 1 microglobulin, fetal growth restriction, haemoglobin, hemopexin

## Abstract

**Objective:**

To assess fetal circulating free fetal haemoglobin (fHbF) levels and heme defences, correlated to fetal circulatory biometry and fetal sex in severe early‐onset fetal growth restriction (FGR).

**Design, Setting and Population:**

A prospective study severe early‐onset fetal growth restriction pregnancies with close clinical management (estimated fetal weight (EFW) < 3rd centile and < 600 g at 20–26 + 6 weeks; *N* = 20).

**Method & Main Outcome Measures:**

Temporal fetal vascular obstetric biometry was recorded. Cord blood fHbF and key heme‐scavenger defences were measured and compared with normal term births (*N* = 26) and births with late‐onset FGR (*N* = 12).

**Results:**

fHbF was elevated in early‐onset FGR compared with normal pregnancy: 0.437(0.337/0.753) mg/mL; and 0.098 (0.045/0.264) mg/mL, respectively (*p* < 0.0001); whilst hemopexin was downregulated in early‐ (*p* < 0.001) and late‐onset FGR (*p* < 0.0001), compared to normal pregnancy: 36(14/81) μg/mL, 25(19/40) μg/mL, and 155(132/219) μg/mL, respectively; median (interquartile ranges). Early‐onset FGR male fetuses had higher HbF compared with the normal males: 0.710(0.433/0.857) mg/mL; (*p* < 0.001); 0.099(0.043/0.246) mg/mL, respectively; median (interquartile ranges). In early‐onset FGR, ratios of mid‐cerebral artery and umbilical artery pulsatility indices correlated positively with heme‐scavenger levels (hemopexin and a heme‐handling composite measure: *p* < 0.05, *r* = 0.672; and *p* < 0.01, *r* = 0.620; respectively), indicating lower levels are associated with cerebral vascular redistribution. These heme handling measures also positively correlated with gestational age at delivery (*r* = 0.713 and *r* = 0.642, respectively, *p* < 0.01, both) and birthweight (*r* = 0.742, *p* < 0.001; and *r* = 0.523, *p* < 0.05; respectively).

**Conclusion:**

Overproduction of fHbF and an inadequate heme defence may contribute to fetal distress and poor umbilical arterial Dopplers in early onset FGR due to elevated placental vascular resistance and vascular inflammation.

## 1 Introduction

We evaluated the occurrence of cell free haemoglobin in the fetal circulation of severe early‐onset fetal growth pregnancies, and measured the autologous heme‐handling capacity, to determine whether they are linked to fetal vascular biometry, indicative of survival risk.

Fetal growth restriction (FGR) represents a failure of a fetus to meet its genetically determined growth potential. Being born small is associated with stillbirth and a host of vascular, neuro‐developmental and endocrine conditions in later life, including, hypertension, diabetes, stroke, coronary artery disease, renal injury and cognitive impairment [1, 2, 3, 4, 5, 6, 7, 8, 9].

Increased extracellular haemoglobin in plasma has been implicated in numerous diseases [10, 11, 12], and vascular dysregulation conditions [13, 14]. Pertinently, high free haemoglobin overcomes the sequestering capacity of plasma defences [15], with deleterious sequalae affecting vascular health [16, 17, 18, 19, 20, 21, 22, 23, 24].

There is excess free fHbF in FGR neonatal cord blood at late preterm or term gestations [24]. We have also demonstrated that fHbF increases fetoplacental vascular resistance by sequestering nitric oxide (NO), promoting matrix damage to the placental stroma, compromising placenta barrier function [24, 25]. We also reported compromised effects of fHbF on placental endothelial junctions in the pro‐inflammatory and pro‐angiogenic effects [24].

The circulating scavengers, haptoglobin, hemopexin and alpha‐1‐microglobin (A1M) protect against fHbF. These endogenous substances are pleiotropic, but within the context of this study, bind fHbF to protect against the sequestration of endothelial‐derived nitric oxide and prevent fHbF from binding to the inflammation‐evoking TLR4 receptor [26, 27], which is strongly expressed in the placenta [28], which activates to elicit expression of numerous inflammatory cytokines placental in a primary trophoblast cell line [29]. Normally, small amounts of fHbF are efficiently handled by these systems with limited impact on vascular function. However, in haemolytic conditions, where there is increased erythrocyte turnover, including megaloblastoma, excess free Hb occurs, either oversaturating existing defences, or leading to their compensatory upregulation [30, 31, 32, 33].

The EVERREST cohort registry is a multi‐centre European prospective cohort study of severe early onset fetal growth restriction (> 3rd centile, by 26.6 weeks of gestation) evaluating the clinical and biological characteristics of pregnancies affected by this condition, [34, 35, 36]. All women were then followed up with growth and Doppler velocimetry surveillance at regular time periods (not > 2 weeks apart), where Doppler abnormalities persist and indicate the need to consider delivery (increased in MCA pulsatility, loss of A wave on ductus venosus assessment, abnormal umbilical artery waveforms), the timeframe between scan surveillance may be < 48 h. Of particular interest, the middle cerebral artery to umbilical artery (MCA/UA) Doppler pulsatility indices (PI) ratio, or cerebroplacental ratio, reflecting cerebral blood redistribution under conditions of placental stress (brain sparing) was assessed. A decreasing MCA/UA ratio is an early and stable biomarker associated with stillbirth risk [37]. A progression towards elevated ductus venosus PI, or absent or reversed UA end diastolic flow waveforms, were also considered as predictors of imminent fetal demise [38, 39, 40].

We hypothesised that fHbF is elevated in cases of early‐onset FGR, with reduced heme scavenging, and is associated with clinical features of fetal blood flow redistribution. Previous observations in PE have demonstrated sexual dimorphism in response to biological challenges [41], which we further explored in early‐onset FGR.

## 2 Methods

Inclusion criteria were singleton live fetuses recruited between 20 + 0 and 26 + 6 weeks where the estimated fetal weight (EFW) on routine anomaly scan was deemed to be both < 3rd centile and < 600 g. The study excluded multiple pregnancies; young maternal age < 18 years; known fetal or congenital abnormalities; maternal infection (HIV, hepatitis); premature rupture of membranes; and where urgent delivery was indicated. None of the participant patients were treated therapeutically with any growth agent, anticoagulant or vascular therapy. A biobank of maternal and fetal cord blood samples was collected, and subjected to biochemical assessment.

Fetal cord blood samples were collected from the umbilical vein of livebirths within a section of the umbilical cord close to the cord insertion point using a syringe and a 21Gauge needle and then into EDTA preservative according to the EVERREST protocol within 30 min of birth. After centrifugation (600× g × 10 min at 4°C), plasma was stored at −80°C within 2 h of delivery [35].

There were two comparison groups for the EVERRST cohort; a normal term group and a late onset FGR group. In the normal group, plasma cord samples were obtained from uncomplicated healthy pregnancies at term, determined by the GROW customised centile method [42] (individualised birthweight centile (IBC) ≥ 10; obtained at St Mary's Hospital, University of Manchester (UoM): citrated tubes; and also at University College London Hospital: EDTA tubes). A late onset FGR group (delivered between 29 and 41 + 1 weeks), defined as IBC ≤ 5 using the GROW algorithm [42], was recruited for plasma cord samples (UoM: citrate tubes). All participants gave written informed permission for inclusion in the study and data to be stored and used for study purposes (REC references: 08/H1010/05, 18/NW/0451, 15/NW/0829, 13/LO/1254).

### 2.1 Sample Analysis

Cord plasma samples were analysed for fHbF and A1M using sandwich ELISAs developed in house [43]. Hemopexin was assayed according to a commercially available assay following the manufacturers protocol (Geneways Bio). Additionally, we used a novel composite score of heme‐handling capacity, whereby the residual heme handling defence levels of A1M and hemopexin were cumulatively considered against prevalent levels of fHbF present in the plasma (sum of hemopexin and A1M as the numerator, accounting for plasma fHbF levels as the denominator).

### 2.2 Obstetric Biometry

Antenatal velocimetry measurements were taken across four clinical sites (University College London Hospital, UK; Skåne University Hospital, University of Lund, Sweden; University Medical Centre Hamburg‐Eppendorf, Hamburg, Germany; and Maternal‐Fetal Unit Hospital Clinic Barcelona, Spain) accordingly to mutually developed standard operating procedures [35].

The Doppler velocimetry PI measurements of UA, MCA and DV reported relate to the last scans preceding delivery; in many cases Caesarean section occurred on the same or following day, according to the best practice outlined by the International Society of Ultrasound in Obstetrics and Gynaecology. Clinical decision for delivery < 34 weeks gestation was made on clinical judgement, but generally in the presence of abnormal umbilical artery waveforms (absent/reversed end diastolic flow), deterioration in MCA Doppler, or absent or reversed flow during atrial contraction in the DV. In such cases all compromised fetuses were delivered by Caesarean section.

### 2.3 Study Groups

Groups of interest were: (1) Early onset‐FGR from the EVERREST cohort; (2) late‐onset diagnosed FGR (> 32 weeks) cases; and (3) normal uncomplicated term pregnancies: unmedicated, in the absence of cardiovascular disease, diabetes and renal disease.

### 2.4 Statistics

Based on our previous study looking at fHbF differences in cord blood samples between a late‐onset FGR and an appropriate for gestational age control group [24], with an 80% power to detect a difference achieving a probable difference of 0.05, we calculated that 12 patient volunteer samples would be required in each group. Kruskal‐Wallis with Dunn's post hoc analysis was used to assess for differences in all analytes between groups. For vascular performance measures, Spearman's rank correlation coefficients were used to explore the relationship between analyte levels and MCA/UA values as a clinical marker of interest. As velocimetry values are gestation‐dependent, *z*‐scores were used according to recognised gestational‐specific reference ranges, to adjust for gestation at time of delivery. Furthermore, early‐onset FGR pregnancies were sub‐grouped according to ductus venosus PI values, obtained most recently to delivery, as: (i) ≤ 94th centile, and (ii) ≥ 95th centile or with loss of DV a‐waves; and their heme‐handling capacities were compared using a Mann–Whitney *U*‐test. Data was expressed as medians ± interquartile range throughout. *p* < 0.05 was considered statistically significant. Data were analysed using Prism (GraphPad Software, Ca, USA).

### 2.5 Raw Data

All raw data are available https://doi.org/10.48420/25924327.

### 2.6 Patient Involvement

Patients were consulted during development of the EVERREST prospective study protocol and participated in a qualitative study to assess their experience of taking part [44].

## 3 Results

Demographics are given in Table S1.

### 3.1 FHbF and Heme Scavenging

**FIGURE 1:**
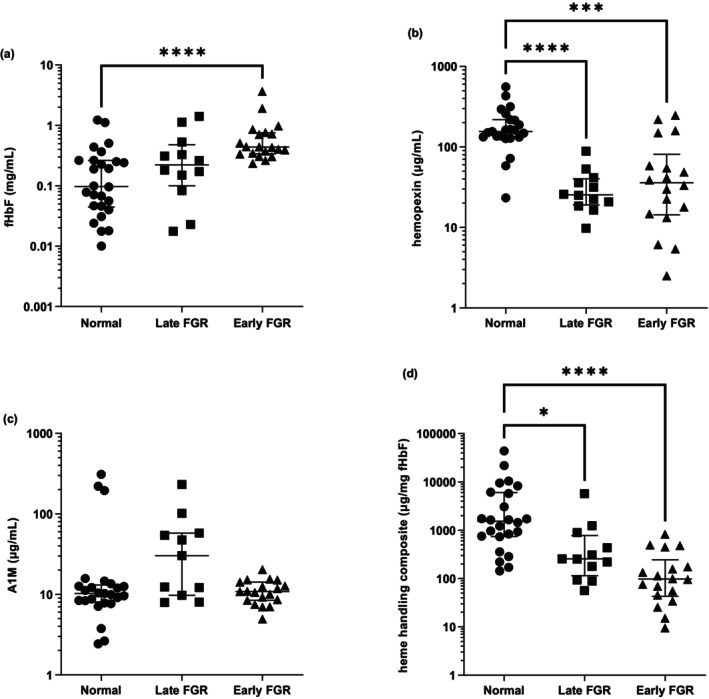
Analyte cord plasma concentrations at delivery. (a) Free fetal haemoglobin, (b) hemopexin and (c) A1M in normal (closed circles), late‐onset FGR (closed squares) and early‐onset FGR (closed triangles). (d) Sample heme‐handling composite scores, derived as (μg/mL hemopexin + μg/mL A1M)/μg/mL fHbF. Kruskall Wallis: (a) *p* < 0.0001; *F*‐test of variance between early‐ and late‐onset FGR: *p* < 0.05. (b) *p* < 0.0001 and (c) N/S; (d) *p* < 0.0001. Dunn's multiple comparison test: **p < 0.05, ***p < 0.001, ****p < 0.0001. Medians (25th centile and 75th centile) are shown.

FHbF levels were higher in the fetal plasma of early‐onset FGR pregnancy compared to normal pregnancy (Figure 1a; 0.437 (0.337,0.753) mg/mL; 0.098 (0.045,0.264) mg/mL; late‐onset FGR levels (0.222 (0.100,0.480) mg/mL) were not significantly different to normal levels; median (25th and 75th percentiles), respectively). Fetal plasma hemopexin levels were lower in early‐onset and late‐onset FGR pregnancies compared to normal pregnancy (Figure 1b; 36 (14,81) μg/mL; 25 (19/40) μg/mL; 155 (132219) μg/mL, respectively; median (25th and 75th percentiles)). The A1M fetal plasma levels were not different between groups (Figure 1c); normal: 10 (8,13) μg/mL; late‐onset FGR: 30 (10,58) μg/mL; early onset FGR: 11 (8,14) μg/mL; median (25th and 75th percentiles). A composite measure of heme handling, accounting for the prevalence of fHbF, was devised, showing that early‐onset FGR fetuses had a statistically significant lower capacity to handle fHbF than normal term fetuses (Figure 1d; 97 (43244); 256 (1361066); 1420 (6836007) μg/mg; respectively; median (25th and 75th percentiles)).

### 3.2 Sex‐Specific Profiles of fHbF and Heme Scavenging

**FIGURE 2:**
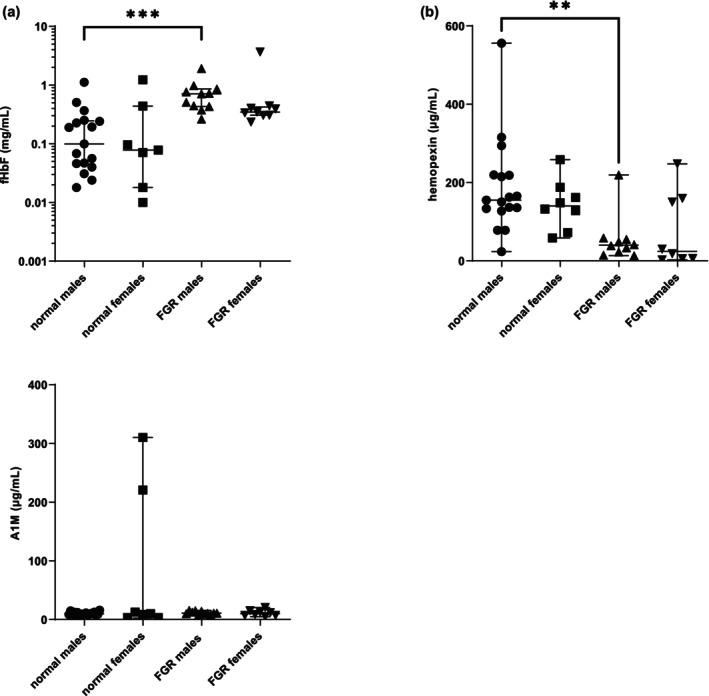
Sex‐specific profiles of free haemoglobin and heme handling proteins in male and female cord blood at delivery from early onset FGR (EVERREST) and normal term pregnancies. (a) Free fetal haemoglobin (fHbF): Kruskal Wallis *p* = < 0.01; Dunn's post hoc test: ***p < 0.001; (b) hemopexin: Kruskal Wallis: *p < 0.05; Dunn's post hoc test: **p < 0.01; (c) A1M: Kruskal Wallis N/S. Medians (25th centile and 75th centile) are shown.

There was a difference in fHbF levels across all normal/early‐onset FGR data split according to fetal sex (Figure 2a; Kruskall Wallis: *p* < 0.001). This was attributable to higher levels among males from early‐onset FGR compared to males from normal pregnancies: 0.710 (0.433,0.857) and 0.099 (0.043,0.246) mg/mL, respectively; Dunn's post hoc: *p* < 0.05; median (25th and 75th percentiles). This group difference was not evident for females: 0.347 (0.306,0.423) and 0.078 (0.018,0.439) mg/mL, respectively; median (25th and 75th percentiles).

There was a difference in hemopexin levels across all normal/early‐onset FGR data split according to fetal sex (Figure 2b; Kruskall Wallis: *p* < 0.01). This was attributable to lower levels among males from early‐onset FGR compared to males from normal pregnancies: 40 (21,56) and 155 (130219) μg/mL, respectively; hemopexin cord blood levels were not different between FGR and normal females groups: 24 (6157) and 140 (86181), respectively, (Dunn's post hoc: *p* < 0.01; median (25th and 75th percentile)).

There were no differences in A1M levels across groups split according to fetal sex (Figure 2c; normal males: 9.6 (8.4,12.2) μg/mL; normal females: 10.0 (12.6220.5) μg/mL; early‐onset FGR males: 10.9 (9.3,14.2) μg/mL; early‐onset FGR females: 10.1 (7.0,14.5) μg/mL; median (25th and 75th percentiles); Kruskall Wallis: NS).

Although birthweights were not significantly different between male and females in the early‐onset FGR group, the male birthweight median was appreciably lower than the females 890 (6451411) g; and 1185 (5121853) g, respectively; median (25th and 75th percentiles); Mann Whitney test: NS.

### 3.3 Heme Handling Biomarkers Linked‐To and Predicting Adverse Perinatal Outcome in the Early Onset‐FGR Group

**FIGURE 3:**
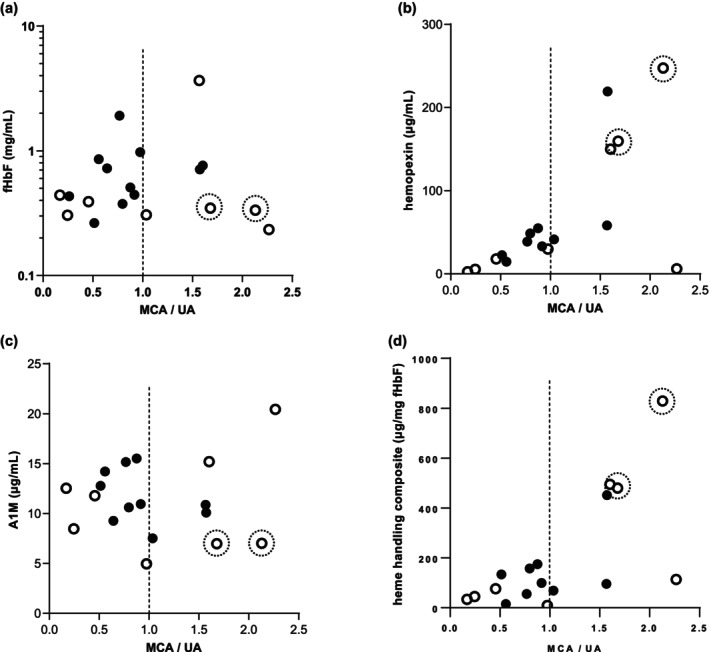
Fetal heme handling associated with clinically relevant ultrasound predictors of adverse perinatal outcome in the EVERREST cohort. Panels depict mid cerebral artery/umbilical artery (MCA/UA) PI value ratios based on the last ultrasound examination prior to delivery in EVERREST cohort. (a) Free fetal haemoglobin versus MCA/UA: Spearman's correlation: NS; (b) hemopexin versus MCA/UA; Spearman's correlation: *r* = 0.672; (c) A1M vs MCA/UA Spearman's correlation: NS; (d) heme handling quantity composite (sum of μg hemopexin and μg A1M, per mg fHbF) versus MCA/UA; Spearman's correlation: *r* = 0.620. Dashed lines indicate threshold at which cerebral redistribution is considered clinically evident. Males (closed circles) and females (open circles) are represented. Two cases with the highest birthweights are indicated with broken circles.

Hemopexin and a composite measure of heme handling correlated positively with the MCA/UA pulsatility indices ratio (Figure 3b,d). Plasma levels of fHbF alone and A1M alone did not correlate in this way (Figure 3a,c, respectively). It is notable that many MCA/ UA pulsatility ratio values were less than value of “1.0” (Figure 3—dashed vertical lines), which is a clinical threshold of a cerebral blood flow redistribution effect and indicative of enhanced stillbirth risk.

DV PIs were grouped according to normality (< 95th centile), or abnormality (≥ 95th centile or with an abnormal waveform) at their last scan, and showed no significant differences in their fHbF levels or heme‐handling defences (Figure S1).

### 3.4 Heme Handling Relationship With Gestational Age at Delivery and Birthweight

**FIGURE 4:**
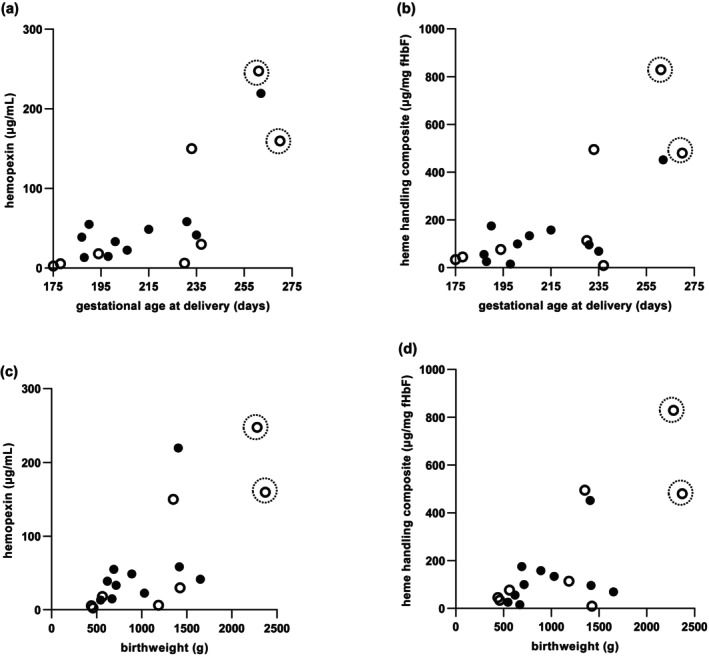
Fetal heme handling directly associates with gestational age and birthweight in the EVERREST cohort. Associations between fetal plasma hemopexin levels in cord blood at delivery and heme handling composite measure with gestational age at delivery (a and b; *r* = 0.713 and 0.642, respectively). Associations between fetal plasma hemopexin levels and heme handling composite measure with birth weight (c and d; *r* = 0.742 and 0.523, *p* < 0.001 and *p* < 0.05, respectively). Males (closed circles) and females (open circles) are represented. Two cases with the highest birth weights are indicated with broken circles.

Both hemopexin and the heme‐handling composite measure correlated significantly with gestational age at delivery (Figure 4a,b) and birthweight (Figure 4c,d). No correlations of any kind were seen in fHbF or A1M (data not shown).

Two females had normal MCA/UA ratios in the highest quartile (Figure 3: broken circles); HbF levels at the lower end of the scale (Figure 3a) and high hemopexin/heme handling levels (Figure 3b,d). They also had the highest birthweights (2730 g and 2280 g respectively; Figure 4) and highest gestational ages at delivery (both at full term: 37 + 2 and 38 + 4 weeks, respectively, compared to a median gestation of 29 weeks).

## 4 Discussion

### 4.1 Main Findings

Fetal cord plasma had significantly higher levels of fHbF in the EVERREST cohort compared to normal pregnancy delivered at term (Figure 1a). We report significant cord plasma reductions in hemopexin in both FGR groups compared to the normal group; being 4–6 times lower in the early‐ and late‐onset groups, respectively, with an apparent inverse relationship to the heme load (Figure 1b). To give further strength to the calculation of heme handling, we derived a composite calculation for each sample (Figure 1d). Using this measure, we found that early‐onset FGR fetal plasma had a lower score than normal term samples (*p* < 0.0001), more significant than for late‐onset FGR (*p* < 0.05). Overall, we did not show any difference between absolute A1M levels in controls and FGR groups, though the inclusion of A1M in the aforementioned composite measure strengthened the separation between early‐ and late FGR cases. The haptoglobin heme‐defence, was below the level of detection in all groups using a manual ELISA method (AbCam, ab219048 Human Haptoglobin SimpleStep ELISA Kit) and by an automated immunoassay analyser method.

Two significant sexual dimorphism findings related only to males: (i) the fetal plasma fHbF level was higher in early FGR pregnancies than occurred normally at term; and (ii) matched by an equally significant reduction in hemopexin (Figure 2).

The final MCA/UA ratio was calculated ahead of Caesarean birth, adjusting for gestation by *z* scoring. For both hemopexin and the composite measure of fHbF handling there were significant positive correlations with MCA/UA (Figure 3b,d, respectively), which indicates that those fetuses with lower heme handling reserve were at greater risk of fetal cerebral vascular redistribution under conditions of placental stress. As an empirical observation, males tended to fall below a MCA/UA value of one, more so than females, indicating a higher mortality risk, (Figure 3a–d).

Correlations of gestational age at delivery and of birthweight against both hemopexin and the composite of heme handling were highly significant, suggesting an improved prognosis for fetuses with higher hemopexin and a higher heme handling composite value. Two cases achieving the highest birthweights (both in excess of 2000 g) in the early‐onset FGR cohort were both female fetuses, and had among the highest hemopexin, high composite measures of heme handling and some of the lowest fHbF levels seen. These cases also achieved the highest gestational ages at delivery (261 and 270 days, versus a cohort median of 203 days), and had high MCA/UA PI levels within the upper group quartile (1.68 and 2.13, respectively), suggesting that improved heme handling may place them at reduced risk.

The DV waveform PI, along with intermittent, absent or reversed end‐diastolic waveforms of UA blood flow are critical predictors of imminent fetal demise, indicating the need to deliver early [38, 39, 40, 45]. Our data suggests that fHbF has no role in the sequalae of events leading to increased resistance from DV blood flow shunts in severe early‐onset FGR.

### 4.2 Interpretation

Our finding of higher fHbF in early‐onset FGR suggests hemolysis is likely a cardinal event, consistent with a global reduction in heme‐handling in FGR. Hypoxia, sensed first in the fetal liver and then the kidney, induces erythropoiesis [46]. Consequently, reticulocytes are larger and more vulnerable than mature red blood cells which make them more likely to lyse contributing to the increased fHbF levels [47]. Our findings complement those of others, who also demonstrated lower circulating hemopexin in cord plasma but in late‐onset FGR group [48]. Previous work has suggested fetal A1M to be lower in FGR than controls [48], implying that this may be an important protective pathway for the foetoplacental endothelium in the context of fHbF exposure [25]. The finding of immeasurable haptoglobin in fetal plasma is in keeping with an understanding of very low levels of haptoglobin in neonatal plasma samples [49].

DV waveform anomalies are likely caused by fetal hypoxaemia‐induced catecholamine release constricting the intrahepatic branches of the portal vein [50], and dysregulated A‐waves are explained through asynchronicity of mitral valve opening in relation to ventricular filling associated with cardiac failure [50]. Our negative finding of an association of heme‐handling analytes with these DV biometry is perhaps unsurprising, as neither of these cardiovascular anomalies implicates the microcirculation, which is the locus of dysregulation caused by fHbF [24].

A MCA/UA ratio below a value of 1.0 is used as a clinical predictor of compensatory fetal cerebral vascular redistribution [51]. A stepwise deterioration in the MCA/UA ratio is commonly used as an early indicator of fetal morbidity associated with poor fetoplacental blood flow, with a vastly increased risk of stillbirth [52]. Positive correlations between hemopexin defence levels and the heme handling composite measure with MCA/UA found in this study suggest a direct effect of fHbF on fetal blood flow redistribution.

Although the mechanisms leading to increased fHbF are incompletely understood, we suggest that once fetoplacental vasoconstriction is evoked by fHbF, fetal hypoxia gradually worsens, initiating a self‐amplifying vicious circle with increased fetal production of HbF and placental overproduction of alpha‐globulin, mediating structural and vascular damage (Figure S1).

These FGR data on prematurity of delivery in early‐onset FGR males, contrast with early‐onset PE where it is known that female fetuses are overrepresented [41]. An epidemiological study suggests male fetuses carry a higher stillbirth risk than females [53]. We suggest further work on the evaluation of sex specific differences in fHbF or heme handling is needed.

Our EVERREST cohort has a similar gestational age profile with the TRUFFLE‐study, since less than 10% of babies were born after 34 weeks of gestation [54]. As with our study, it is likely that a range of aetiologies are operative in causing FGR, including double‐stressor hits. The onset times for different pathologies linked to FGR, including elevations in fHbF, might vary in different pregnancies, implying differences in gestational longevity. Disease onset might also be concomitant with severity of effect. Considering fHbF as one important vascular stressor, our previous work using ex vivo perfused healthy human placentas and in vitro placental endothelial cultures, found effects on fetoplacental vasoconstriction and endothelial inflammation, which were dose dependent for exogenous fetal haemoglobin [24]. In a previous study, we also found that fHbF was elevated in the maternal circulation in preeclampsia, a disease associated with FGR [55]. In the TRUFFLE study, maternal hypertension was a strong associated factor with FGR, which we speculate might partly be explained by a vasoconstriction effect of elevated fHbF in this circulation.

Our data in this study suggests the haemoglobin effect is only on the microcirculation, as our MCA:UA ratio was unaffected, whilst umbilical arterial Doppler velocimetry was impacted. In common with some cases in the TRUFFLE study, we propose a common pathway exists, where FGR and preeclampsia and associated with placental hypoxia/oxidative stress. This evokes fetal megablastic erythropoiesis with consequential hemolysis and unchecked free haemoglobin levels (Figure S2). It is yet to be determined whether the in utero exposure to high levels of free haemoglobin, common to the full fetal circulation, might impact on the health of organs of babies born at term into the neonatal period.

### 4.3 Strengths and Limitations

The EVERREST cohort is an extensively characterised, data‐rich, prospective European registry, comprising severe early‐onset FGR diagnosed in the second trimester (EFW < 3rd centile and < 600 g, 20–26 + 6 weeks of gestation, no known chromosomal, genetic or major structural abnormalities) and representing the severest forms of placental‐driven disease with high rates of perinatal loss [34, 35, 36]. It represents a unique opportunity to evaluate fHbF status and heme handling defences. This definition does not necessarily meet the Delphi definition of early FGR at the point of recruitment [56], but practically included pregnancies at high risk of FGR. In hindsight all babies were born below the 3rd centile (16/20 cases at zero), with or without evidence of placental insufficiency, reducing the chance inclusion of SGA or constitutionally small fetuses. Due to the time of recruitment being around the publication time of the Delphi criteria for FGR, the late onset FGR inclusion criteria were based on IBCs ≤ 5th centile, which might have weakened an ability to find differences in cord blood fHbF levels between the normal and late‐onset FGR groups.

A clear limitation of the study is the inability to track longitudinal readings of heme levels and its defences in fetal plasma across gestation. This would be best achievable in an in vivo animal model, where an elevated fetal heme status could be employed.

Whilst inclusion of a preterm gestational cohort would have been ideal, we chose not to include it, since all of our early‐onset FGR cases were delivered by Caesarean section, and an appropriate, healthy, well grown premature control group delivered by Caesarean was unavailable, since most preterm births are confounded by other conditions such as infection or placental abruption. However, in support of likely vascular dysregulation from unchecked free haemoglobin in the fetal circulation in early pregnancy, the TLR4 receptor, which binds free haemoglobin [27], is present in the first trimester placenta [28] and continues with high expressed levels across the second trimester until term [57]. Toll‐like receptors evoke a danger response in vascular and other systems, initiating inflammation in response to cellular stresses [58].

## 5 Conclusion

We have demonstrated that fHbF is overrepresented in the severest cases of early onset FGR, with a consequential overwhelming of heme defences, a scenario linked to poor fetal vascular performance, severe foetoplacental insufficiency, and preterm delivery. In the early‐onset FGR group, sex‐specific variations in response to fHbF and heme appear to exist with female fetuses displaying higher MCA PI values and a greater proportion of female fetuses showing MCA/UA ratios in excess of 1, a clinical threshold below which is associated with increased stillbirth risk. Among this group we also observed a tendency for female fetuses to have higher hemopexin levels and more active heme handling profiles.

## Author Contributions

Conceptualization, planning, formal analysis, funding acquisition, project administration, role/writing: A.B., S.R.H., A.L.D. and P.B.; investigation and methodology: A.B., G.B., A.E., C.B.‐R., F.D., Y.G., T.W., J.B., E.H., A.D., K.H., K.M., N.M. and R.N.S.; supervision: P.B., A.D.L. and S.R.H.; review and editing: all authors.

## Ethics Statement

The study was compliant with the Declaration of Helsinki principles. The EVERREST databank was accessed utilising existing ethics approval National Research Ethics Service Committee London—Stanmore in the UK (REC reference: 13/LO/1254). A detailed protocol for this multi‐centre collaboration has been previously published, describing the collection parameters and clinical methodology [35]. Plasma cord samples from uncomplicated healthy pregnancies at term and late‐onset FGR were covered by North West—Haydock Research Ethics Committee (REC references: 08/H1010/55(+5), 18/NW/0451 and 15/NW/0829). Additional plasma cord samples from uncomplicated healthy pregnancies were collected (REC reference: 13/LO/1254). All participants gave written informed permission for inclusion in the study and data to be stored and used for study purposes.

## Conflicts of Interest

The authors declare no conflicts of interest.

## Supporting information


Figure S1.

Figure S2.

Table S1.

## Data Availability

The data that support the findings of this study are openly available in Research data for a study on haemoglobin and heme defences at https://doi.org/10.48420/25924327.
