# Supplementary material for: Free Fetal Haemoglobin in Severe Early‐Onset Fetal Growth Restriction: A Prospective Multi‐Centre Study
Source: BJOG. 2025 Feb 19;133(3):401–11. doi: 10.1111/1471-0528.18104 (PMC12770088; doi:10.1111/1471-0528.18104)
Supplement: Supplementary file 1 — Figure S1. [file BJO-133-401-s001.pptx]

## Slide 1
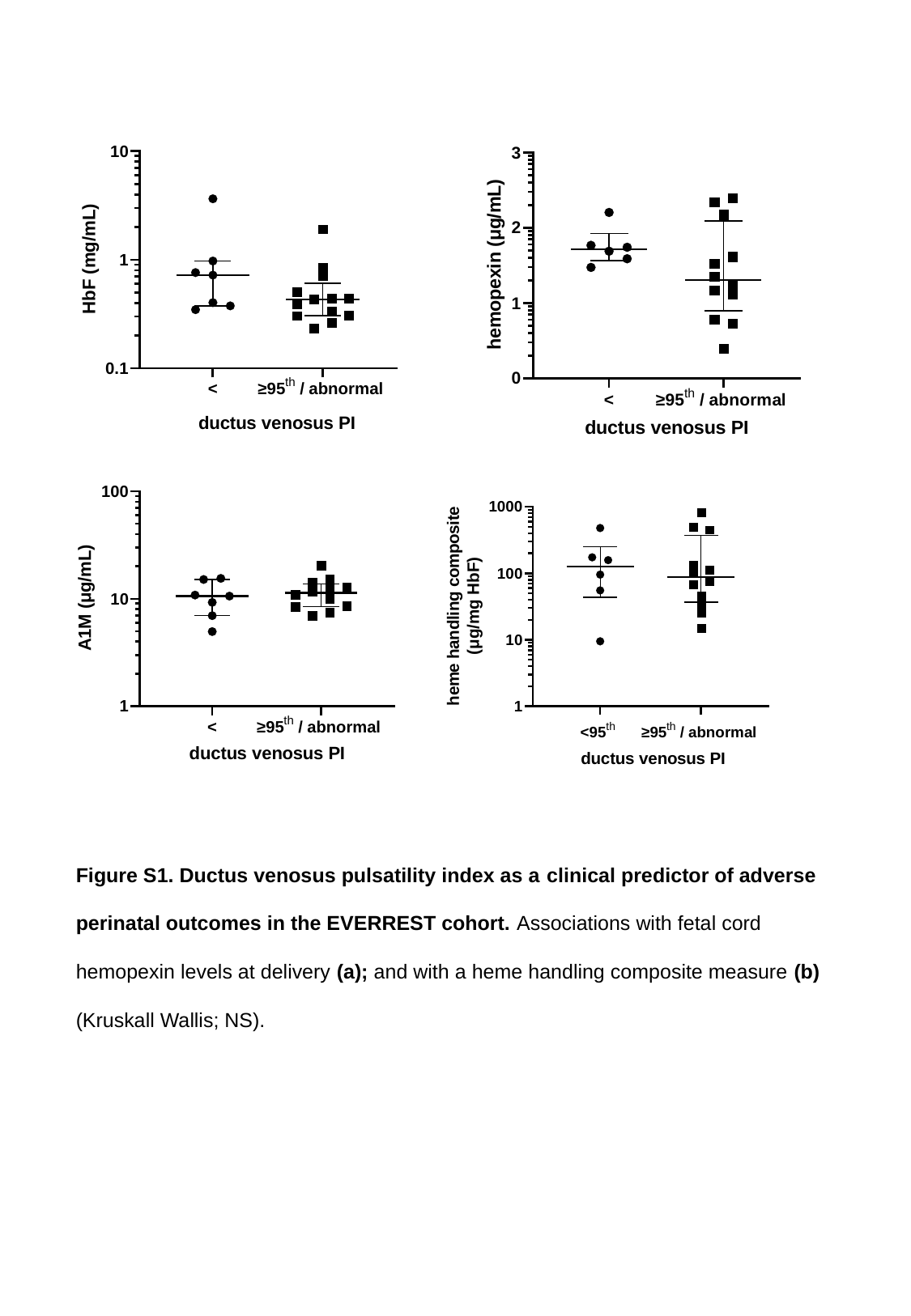

Figure S1. Ductus venosus pulsatility index as a clinical predictor of adverse perinatal outcomes in the EVERREST cohort. Associations with fetal cord hemopexin levels at delivery (a); and with a heme handling composite measure (b) (Kruskall Wallis; NS).
