# Supplementary material for: Free Fetal Haemoglobin in Severe Early‐Onset Fetal Growth Restriction: A Prospective Multi‐Centre Study
Source: BJOG. 2025 Feb 19;133(3):401–11. doi: 10.1111/1471-0528.18104 (PMC12770088; doi:10.1111/1471-0528.18104)
Supplement: Supplementary file 2 — Figure S2. [file BJO-133-401-s003.pptx]

## Slide 1
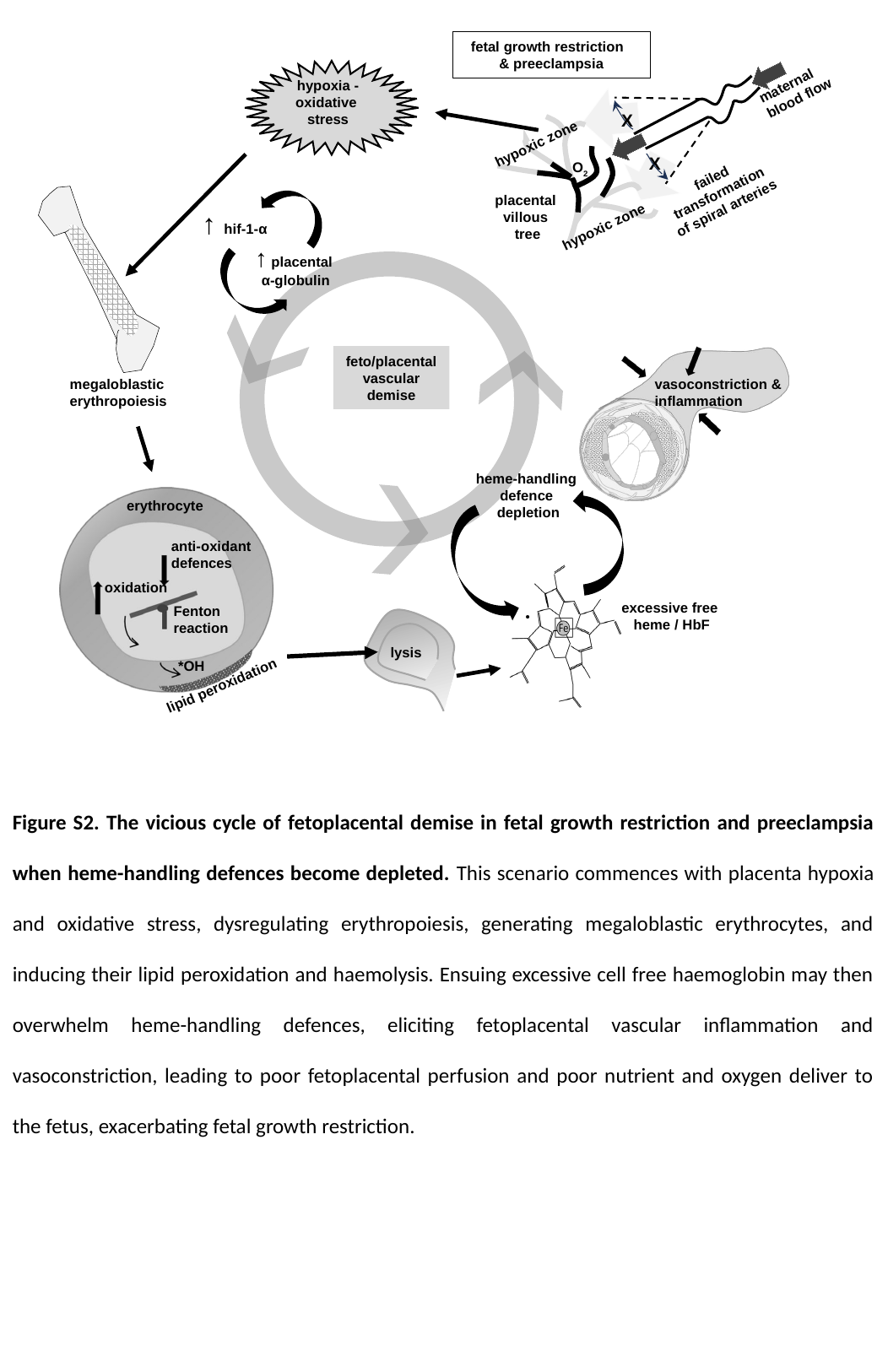

fetal growth restriction
& preeclampsia
hypoxia -
oxidative
stress
maternal
blood flow
X
hypoxic zone
X
O2
failed
transformation
of spiral arteries
placental
villous
tree
hypoxic zone
↑ hif-1-α
↑ placental
α-globulin
feto/placental
vascular
demise
vasoconstriction &
inflammation
megaloblastic erythropoiesis
heme-handling
defence
depletion
erythrocyte
anti-oxidant
defences
oxidation
Fenton
reaction
*OH
lipid peroxidation
excessive free
heme / HbF
lysis
Figure S2. The vicious cycle of fetoplacental demise in fetal growth restriction and preeclampsia when heme-handling defences become depleted. This scenario commences with placenta hypoxia and oxidative stress, dysregulating erythropoiesis, generating megaloblastic erythrocytes, and inducing their lipid peroxidation and haemolysis. Ensuing excessive cell free haemoglobin may then overwhelm heme-handling defences, eliciting fetoplacental vascular inflammation and vasoconstriction, leading to poor fetoplacental perfusion and poor nutrient and oxygen deliver to the fetus, exacerbating fetal growth restriction.
