# Supplementary material for: Free Fetal Haemoglobin in Severe Early‐Onset Fetal Growth Restriction: A Prospective Multi‐Centre Study
Source: BJOG. 2025 Feb 19;133(3):401–11. doi: 10.1111/1471-0528.18104 (PMC12770088; doi:10.1111/1471-0528.18104)
Supplement: Supplementary file 3 — Table S1. [file BJO-133-401-s002.docx]

**Table S1. Demographics**

| Characteristic | Early-onset FGR  n=20 | Late-onset FGR  n=12 | Normal pregnancy  n=26 | P  ANOVA |
| --- | --- | --- | --- | --- |
| Maternal age: years (range) | 32 (19-43) | 29 (22-45) | 33 (24-41) | NS^A^ |
| Ethnicity: n (%)  White  Non-white  Other | 10 (50)  10 (50)  0 (0) | 11 (92)  1 (8)  0 (0) | 15 (58)  1 (4)  10 (38) | <0.0001^B^ |
| Parity: n (%)  0  1  ≥2 | 13 (65)  2 (10)  5 (25) | 4 (33)  8 (66)  0 (0) | 9 (35)  12 (46)  5 (19) | <0.05^B^ |
| Concomitant preeclampsia | 4 | 0 | 0 |  |
| Birthweight: (g)  median (range) | 803 (441-2370)*** | 1960 (654-2660)*** | 3295 (2320-4220) | <0.001^B^ |
| IBC: median (range) | 0 (0-3)**** | 2 (0-5)*** | 50 (10-97) | <0.0001^A^ |
| Gestational age at delivery: median (range) | 204 (175-270)**** | 259 (203-281)* | 274 (253-287) | <.0.0001^A^ |
| Mode of delivery: n (%)  VD  CS | 0 (0)  20 (100) | 3 (25)  9 (75) | 8 (31)  18 (69) | <0.05^B^ |
| Fetal sex: n (%)  Male  Female | 11 (55)  9 (45) | 5 (42)  7 (58) | 16 (62)  9 (35) | NS^B^ |

^A^Kruskall Wallis with Dunn’s post hoc analysis. ^B^Chi-square. * *** ****post-hoc c.f. normal group; ^*^P<0.05 considered significant. #Individualized birth weight centile (IBC) were calculated using Gestation Related Optimal Weight (GROW) software (Perinatal Institute, Birmingham, United Kingdom), as ≤ 5th and ≥10th centile, respectively (1).

1. Gardosi J, Francis A, Turner S, Williams M. Customized growth charts: rationale, validation and clinical benefits. Am J Obstet Gynecol. 2018;218(2s):S609-s18.
